# Pro-inflammatory activation changes intracellular transport of bevacizumab in the retinal pigment epithelium in vitro

**DOI:** 10.1007/s00417-021-05443-2

**Published:** 2021-10-13

**Authors:** Julia Hildebrandt, Tom Käckenmeister, Katrin Winkelmann, Philipp Dörschmann, Johann Roider, Alexa Klettner

**Affiliations:** Department of Ophthalmology, University Medical Center, University of Kiel, Quincke Research Center, Rosalind-Franklin-Str. 9, 24105 Kiel, Germany

**Keywords:** Bevacizumab, Toll-like receptor (TLR-3), Retinal pigment epithelium (RPE), Transepithelial transport, Actin filaments, Myosin 7a

## Abstract

**Purpose:**

Bevacizumab is taken up and transported through the retinal pigment epithelium. Inflammatory signaling may influence this interaction. In the present study, we have investigated the effect of pro-inflammatory stimuli on the uptake, intracellular localization, and transepithelial transport of bevacizumab.

**Methods:**

ARPE-19 cell line or primary porcine RPE cells were treated with clinical relevant concentrations of bevacizumab (250 µg/ml). Pro-inflammatory signaling was induced by TLR-3 agonist polyinosinic:polycytidylic acid (Poly I:C). Viability was investigated with MTT and trypan-blue exclusion assay, and cell number, uptake, and intracellular localization were investigated with immunofluorescence, investigating also actin filaments, the motor protein myosin 7a and lysosomes. Immunofluorescence signals were quantified. Intracellular bevacizumab was additionally detected in Western blot. Barrier function was investigated with transepithelial resistant measurements (TER). The transepithelial transport of bevacizumab and its influence on cytokine (IL-6, IL-8, IL-1β, TNFα) secretion was investigated with ELISA.

**Results:**

Poly I:C in combination with bevacizumab reduced the viability of the cells. Treatment with Poly I:C reduced the uptake of bevacizumab, changed the intensity of the actin filaments, and reduced the colocalization with myosin 7a. In addition, Poly I:C reduced the capacity of RPE cells to transport bevacizumab over the barrier. In addition, bevacizumab reduced the secretion of IL-8 and TNFα after Poly I:C stimulation at selected time points.

**Conclusions:**

Pro-inflammatory activation of RPE cells with TLR-3 agonist Poly I:C changes the interaction of RPE cells with the anti-VEGF compound bevacizumab, reducing its uptake and transport. On the other hand, bevacizumab might influence pro-inflammatory cytokine release. Our data indicate that inflammation may influence the pharmacokinetic of bevacizumab in the retina.


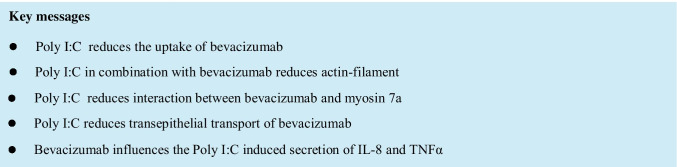


## Introduction

Age-related macular degeneration (AMD) is the main cause for vision loss in the Western world and its prevalence is worldwide on the rise [1, 2]. It presents in an early form, which is asymptomatic for the patient, and two late forms. In the atrophic late form of AMD, the retinal pigment epithelium and, secondarily, the photoreceptors degenerate, causing a slow deterioration of vision. In the exudative late form of AMD, vessels grow from the choroid beneath and into the retina, which cause a fast and severe loss of visual acuity [3, 4]. While AMD is a multifactorial disease [5–7], vascular endothelial growth factor (VEGF) is the main culprit in its exudative form [8]. Therefore, in exudative AMD, anti-VEGF therapy is the state-of-the art treatment [9]. Despite the official approval of anti-VEGF therapies such as ranibizumab or aflibercept for treatment of AMD [10, 11], off-label application of bevacizumab is common due to the lower price and the comparable outcomes [12, 13].

The retinal pigment epithelium (RPE) is an epithelial monolayer situated between the choroid and the photoreceptors. It is vital for the maintenance of vision in the retina with many functions including phagocytosis of photoreceptor outer fragments or cytokine secretion [14]. RPE cells comprise a barrier, shielding the retina from the choroidal blood system, providing a controlled transepithelial transport to and from the retina [15]. In addition, the RPE is important in regards to regulation of the immune response and inflammatory activation of the retina [16]. RPE cells express toll-like receptors (TLR), which are pattern recognition receptors enabling them to react fast to so-called danger signals and contribute to the protection of the retina against these stimuli [17]. TLR activation induces a differentiated cytokine release in RPE cells, but can also result in cell death and VEGF secretion [18–20].

We and others have previously shown that bevacizumab is taken up by the RPE and transported through the epithelial barrier [21–23]. In areas where the RPE barrier is still intact, this may be an important pharmacokinetic mechanism for the anti-VEGF compound to reach its target region, the subretinal space [21–25]. Inflammation is an important part of the pathogenesis of AMD, and in the degenerating retina, danger signals are abundantly present which may activate the remaining RPE [7, 26]. We have previously investigated the effects of pro-inflammatory activation of RPE cells on viability, function, and barrier [20, 27]. The pro-inflammatory activation may influence the interaction of the RPE with anti-VEGF compounds. In the present study, we have investigated the uptake, intracellular presence, and transepithelial transport of bevacizumab in RPE cells under the stimulation of the TLR-3 agonist Poly I:C. In addition, we have analyzed the effect of bevacizumab treatment on pro-inflammatory cytokine release induced by Poly I:C stimulation.

## Material and methods

### Cell culture

The human immortal RPE cell line ARPE-19 was obtained from ATCC. Culture medium consisted of DMEM, supplemented with penicillin/streptomycin (1%), non-essential amino acids (1%), and 10% fetal calf serum. ARPE-19 cells were seeded at a concentration of 100,000 cells per well in 12-well plates for MTT assessment and on collagen-coated cover slips for immunofluorescence assessment.

For experiments concerning transepithelial transport and cytokine release, primary porcine RPE cells were used. They were prepared as described previously [28]. In brief, porcine eyes were obtained from a local slaughter house within 4 h post mortem. The anterior parts, vitreous, and retina were removed and the RPE harvested with trypsin digestion. Cells were cultivated in HyClone DMEM (GE Healthcare (#SH30022.01), München, Germany), supplemented with penicillin/streptomycin (1%; #A2213), HEPES (2.5%; #L1613), non-essential amino acids (1%; #K0293) (all Biochrome, Berlin, Germany), and 10% fetal calf serum (LINARISblue, Wertheim-Bettingen, Germany, #SBF3119KYA). For experiments concerning barrier function (TER measurements), they were seeded on transwell membranes (Sarstedt, #83.3931.041). For experiments testing cytokine secretion, cells were seeded at on 12-well plates (Sarstedt, Nümbrecht, Germany, #83.3921). All experiments were conducted without further passaging and at confluence and, when seeded on transwell membranes, after reaching stable transepithelial electrical resistance.

### Treatment of cells

Cells were treated with 250 µg/ml bevacizumab (Avastin; Roche, Mannheim, Germany), which is considered a clinically relevant concentration [29], for indicated time periods. Pro-inflammatory activation was conducted with TLR-3 agonist polyinosinic:polycytidylic acid (Poly I:C; 1, 10, or 100 µg/ml; Tocris Bioscience, Bristol, UK; #4287/10). Time frame of experiments was 1 to 28 days. Medium was changed twice per week and Poly I:C and bevacizumab were added in the appropriate wells with each medium change.

### TER measurements

Barrier function of the primary RPE cell monolayer was assessed by transepithelial electrical resistance (TER) in primary porcine RPE cells [30]. TER measurement was conducted with an epithelial voltohmmeter (EVOM2; Word Precision Instruments, Sarasota, FL, USA), using STX2-chopstick electrodes. TEER was evaluated using the formula: TER = (measurement − blank value) (Ω) × membrane area (cm^2^).

### MTT assay

MTT assay is an established viability assay [31] and was conducted as previously described [32]. In brief, cells were incubated with 0.5 mg/ml MTT (3-(4,5-dimethylthiazol-2-yl)-2,5-diphenyltetrazoliumbromid; Sigma-Aldrich, #M2128), solved in DMEM without phenol red (GE Healthcare, #SH3028.01), washed, and lysed in dimethyl sulfoxide (DMSO; Roth, Karlsruhe, Germany, #7029.1). Absorption was measured at 550 nm with a spectrometer (Elx800, BioTek, Bad Friedrichshall, Germany).

### Trypan-blue exclusion assay

Cell viability was determined by trypan blue exclusion assay as previously described [33]. Cells were briefly washed with PBS (with penicillin and streptomycin) and incubated for 5 min with trypsin–EDTA at 37 °C. Cell culture medium as was applied and cells were suspended in a final volume of 1 ml. Of these, 20 µl were suspended in 20 µl trypan blue (Sigma, Deisenhofen), and cells were counted in a Neubauer cell chamber.

### ELISA

In order to assess the transepithelial transport of bevacizumab, it was added to the apical chamber of a transwell plate, harboring primary RPE cells with stable barrier function. The amount of bevacizumab in the lower chamber was assessed after 1 h using an IgG ELISA (eBioscience, Frankfurt, Germany) as previously described [21]. In brief, a microtiter plate was coated with capture antibody, washed and blocked, and filled with 100-µl samples or standard, and incubated for 2 h. After washing, the detection antibody was added at room temperature for 1 h and after additional washing, the substrate solution was added and the reaction stopped after 15 min using a stop solution. The plate was then analyzed at 450 nm with a spectrometer (Elx800; BioTek, Winooski, Vermont, USA). For testing the effect of bevacizumab on cytokine secretion, porcine specific kits from R&D systems were used and conducted according to the manufacturer’s instructions (IL-6 (#P6000B), IL-1β (#PLB00B), IL-8 (#P8000), TNFα (#PTA00)). These ELISAs were conducted with supernatants harvested from primary RPE cells, treated for the indicated time period with the indicated stimulus.

### Immunofluorescence

Immunofluorescence was conducted in order to determine the uptake of bevacizumab and its colocalization with actin filaments, myosin 7a and the lysosomal marker Lamp2. This was done as previously described with modification [24]. ARPE-19 cells were seeded on cover slips (Th. Geyer) coated with Collagen A (Biochrome). Confluent cells were treated with 250 µg/ml bevacizumab and/or with Poly I:C (1, 10, or 100 µg/ml) for different time intervals (1 day, 7 days, 28 days). After treatment, cells were washed, permeabilized, blocked, and incubated with primary antibodies (Anti-IgG, Sigma Aldrich, I-1011, 1:1000; anti-Myosin7a (H60), Santa Cruz, sc-25834, 1:200; Lamp2 (C-20), Santa Cruz, sc-8100, 1:200) for 1 h at room temperature in a humid chamber. Cells were washed and a corresponding secondary antibody (Alexa Fluor 555 goat-anti-human IgG, 1:1000; Alexa Fluor 647 donkey-anti-rabbit, Invitrogen, 1:500; or Alexa Fluor 647 donkey-anti-goat, Invitrogen, 1:500) was added for 1 h at room temperature, including 0.4 µM bisbenzimide H (Sigma-Aldrich) for nucleus staining. To visualize actin filaments, Atto488-phalloidin (Sigma Aldrich) was added in some experiments. Cover slips were mounted and examined using Zeiss Imager.M2 microscope together with the Zeiss ApoTome. The microscope was fitted with the Zeiss AxioCam MRm Camera (Carl Zeiss Microscopy GmbH, Germany). All images were analyzed using Axio-Vision and Zen Software by Zeiss (Carl Zeiss). Cell nuclei and number of events for each stain were quantified, and the relative intensities were normalized. Quantification was done with Fiji Software (ImageJ-win64; https://imagej.net/Fiji). To quantify the immunofluorescence images, they first were split into their single wavelengths. Each of those newly created images was converted into a monochrome picture. After the background was subtracted, the number of events was automatically counted by the program. Each experiment was repeated for at least three to six times and six images were taken per cover slip.

### Western blot

Intracellular bevacizumab was additionally investigated in Western blot as described previously [21, 24]. In brief, after treatment with bevacizumab for indicated time periods, cells were lysed according to standard protocol. Proteins were separated on a standard SDS-PAGE, blotted on PVDF membrane, and treated with the appropriate primary antibody (Anti-IgG, 1:1000, Sigma Aldrich I-1011; anti-β actin, 1:1000, Rockland, #600–401-886) overnight, followed by the appropriate HRP-conjugated secondary antibody (Cell Signaling Technology). The signal was detected with MF-ChemiBis 1.6 (Biostep, Jahnsdorf, Germany).

### Statistics

Each experiment was independently repeated at least three times. Number of experiments assessed is given in the respective figure legends. Diagrams depict mean and SD. Statistical analysis was conducted with Student’s *t* test. A *p* value of ≤ 0.05 was considered significant.

## Results

### Viability

**Fig. 1:**
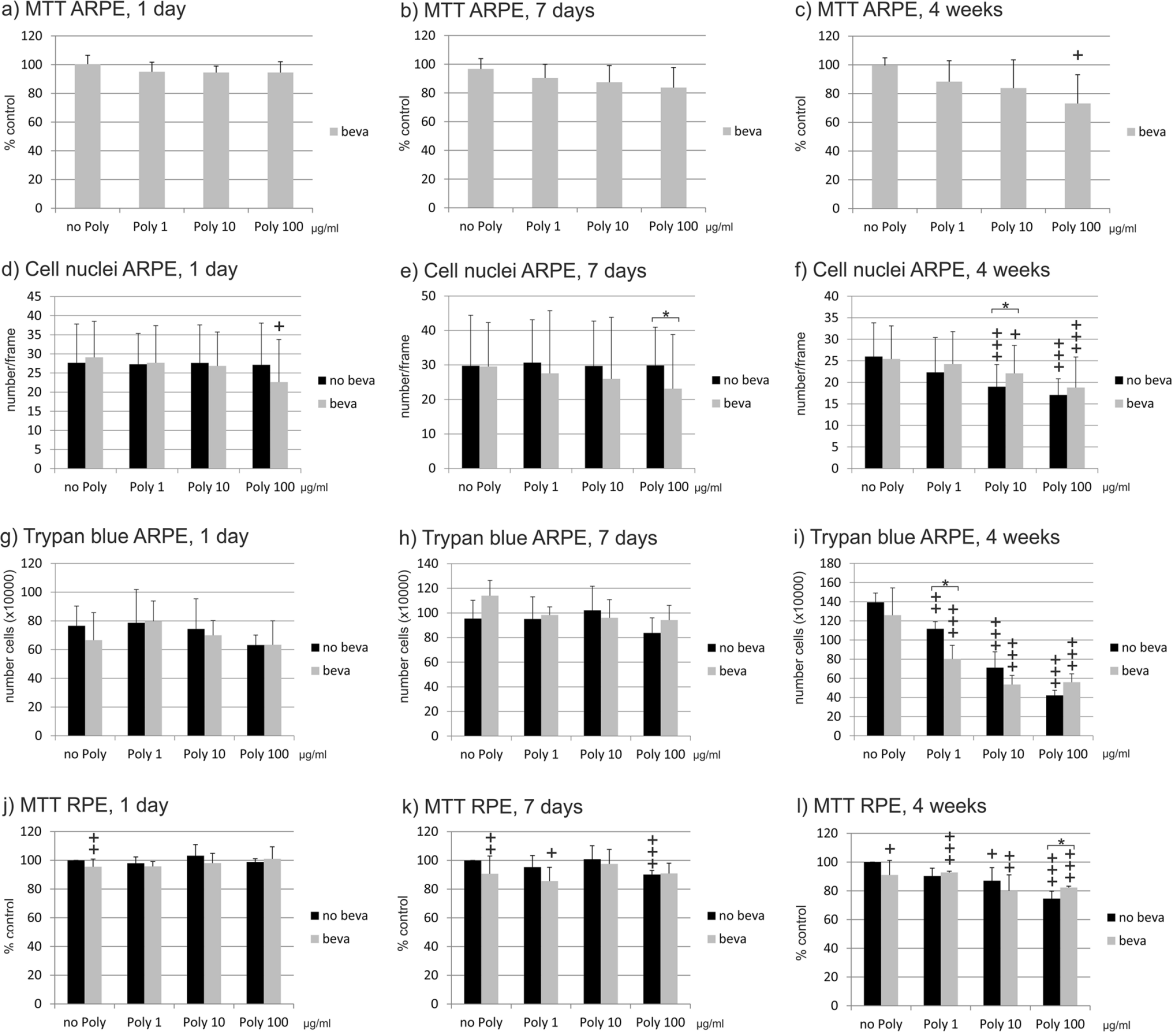
Viability of ARPE-19 cells treated with 250 µg/ml bevacizumab and 1, 10, or 100 µg/ml Poly I:C measured in MTT (**a**–**c**), by number of cell nuclei (**d**–**f**), or by trypan-blue exclusion assay (**g**–**i**) treated with or without bevacizumab and 1, 10, or 100 µg/ml Poly I:C (**d**–**f**) for 1 day (**a**, **d**, **g**), 7 days (**b**, **e**, **h**), or 4 weeks (**c**, **f**, **i**). In addition, the effect on the viability was tested in primary porcine RPE cells (**j**–**l**). Treatment for 4 weeks with Poly I:C and bevacizumab results in a significant loss of cell viability of ARPE-19 cells in MTT (**c**). Treatment for 1 day with 100 µg/ml Poly I:C and 250 µg/ml bevacizumab significantly reduced the number of cell nuclei (**d**). After 7 days, the number of nuclei of cell treated with bevacizumab and 100 µg/ml Poly I:C is significantly lower than when treated with bevacizumab alone (**e**). After 4 weeks, both 10 and 100 µg/ml Poly I:C with or without bevacizumab reduce the number of nuclei (**f**). In trypan-blue exclusion assay, any treatment for 1 or 7 days did not change the number of cells. After 4 weeks, Poly I:C at any concentration with or without bevacizumab reduced the cell number significantly. At Poly I:C 1 µg/ml, the addition of bevacizumab reduced the cell number compared to treatment with Poly I:C alone. In primary RPE cells, treatment with bevacizumab reduced cell viability at each time point tested (**j**–**l**). After 7 days, viability is reduced after treatment with Poly I:C 1 µg/ml and bevacizumab and with Poly 10 alone (**k**). After 4 weeks of treatment, Poly I:C with 10 and 100 µg/ml alone, and Poly I:C with bevacizumab at all concentrations reduced viability compared to control. The difference between Poly I:C 100 μg/ml with and without bevacizumab is significant with a higher viability with additional treatment with bevacizumab. Statistical significance evaluated with Student’s *t* test. + *p* < 0.05, +  + *p* < 0.01, +  +  + *p* < 0.001 compared to control; *p < 0.05 comparing treatment with or without bevacizumab (**a**–**c**, *n* = 3; **d**–**f**, *n* = 36; **g**–**i**, *n* = 4–5; **j**–**l**, *n* = 4–13). Abbreviations: beva = bevacizumab, Poly = polyinosinic:polycytidylic acid

ARPE-19 cells were treated for 1 day, 7 days, and 4 weeks (28 days) with 250 µg/ml bevacizumab and/or 1, 10, or 100 µg/ml Poly I:C. Treatment of ARPE-19 cells with any stimulus in any combination did not exert any effect on cell viability after 1 or 7 days (Fig. 1a, b). However, 100 µg/ml Poly I:C with bevacizumab significantly reduced cell viability (Fig. 1c). We have shown before that the treatment with 100 µg/ml Poly I:C also reduced the viability [20] and the differences between cells treated with or without bevacizumab are not significant.

In addition, we have evaluated the number of cell nuclei in relation to the treatment in immunofluorescent microscopy. After treatment for 1 day, the combined treatment of ARPE-19 cells with 100 µg/ml Poly I:C and 250 µg/ml bevacizumab significantly reduced the number of cell nuclei compared to control (Fig. 1d). After 1 week of stimulation, the combined treatment with 100 µg/ml Poly I:C and 250 µg/ml bevacizumab showed a significant difference between cells treated with 100 µg/ml Poly I:C with or without bevacizumab (Fig. 1e). After 4 weeks, the treatment with 10 and 100 µg/ml Poly I:C with or without bevacizumab reduced the number of cell nuclei significantly (Fig. 1f). The difference with or without treatment with bevacizumab is significant for 10 µg/ml Poly I:C. In addition, we investigated the influence of Poly I:C with and without bevacizumab on the cell number in a trypan-blue exclusion assay. The cell number of ARPE-19 cells is not influenced by treatment with any concentration of Poly I:C after 1 day and 7 days (Fig. 1g, h), but displays a significant reduction after 4 weeks at any Poly I:C concentration tested (Fig. 1i). Of note, Poly I:C displays a concentration-dependent effect with a significant decrease of cell number between the different concentrations. Similarly, combined stimulation with Poly I:C at any concentration with bevacizumab significantly reduces the cell number, with a combined treatment of 1 µg/ml Poly I:C with bevacizumab significantly decreasing the cell number compared to Poly I:C 1 µg/ml alone (Fig. 1i).

Also, we have evaluated the effect of Poly I:C in combination with bevacizumab on primary RPE cells. Our data show a decrease of viability of cells treated with bevacizumab alone compared with untreated control at all time points tested (Fig. 1j–l). Treatment with Poly I:C with or without bevacizumab did not display any decrease in viability after 24 h (Fig. 1j). After 7 days, treatment with 100 µg/ml Poly I:C and the combined treatment with 1 µl/ml Poly I:C and with bevacizumab decreased cell viability (Fig. 1k). After 4 weeks, treatment with 10 or 100 µg/ml Poly I:C as well as Poly I:C in all concentrations combined with bevacizumab significantly reduced cell viability (Fig. 1l). Of note, at 100 µg/ml Poly I:C, viability in combination with bevacizumab is significantly higher than with 100 µg/ml Poly I:C alone (Fig. 1l).

Taken together, our data show that pro-inflammatory activation with Poly I:C can reduce cell viability, especially when treated for extended periods of time, and that additional treatment with bevacizumab may influence this effect, depending on the time frame and cell type.

### Intracellular uptake

**Fig. 2:**
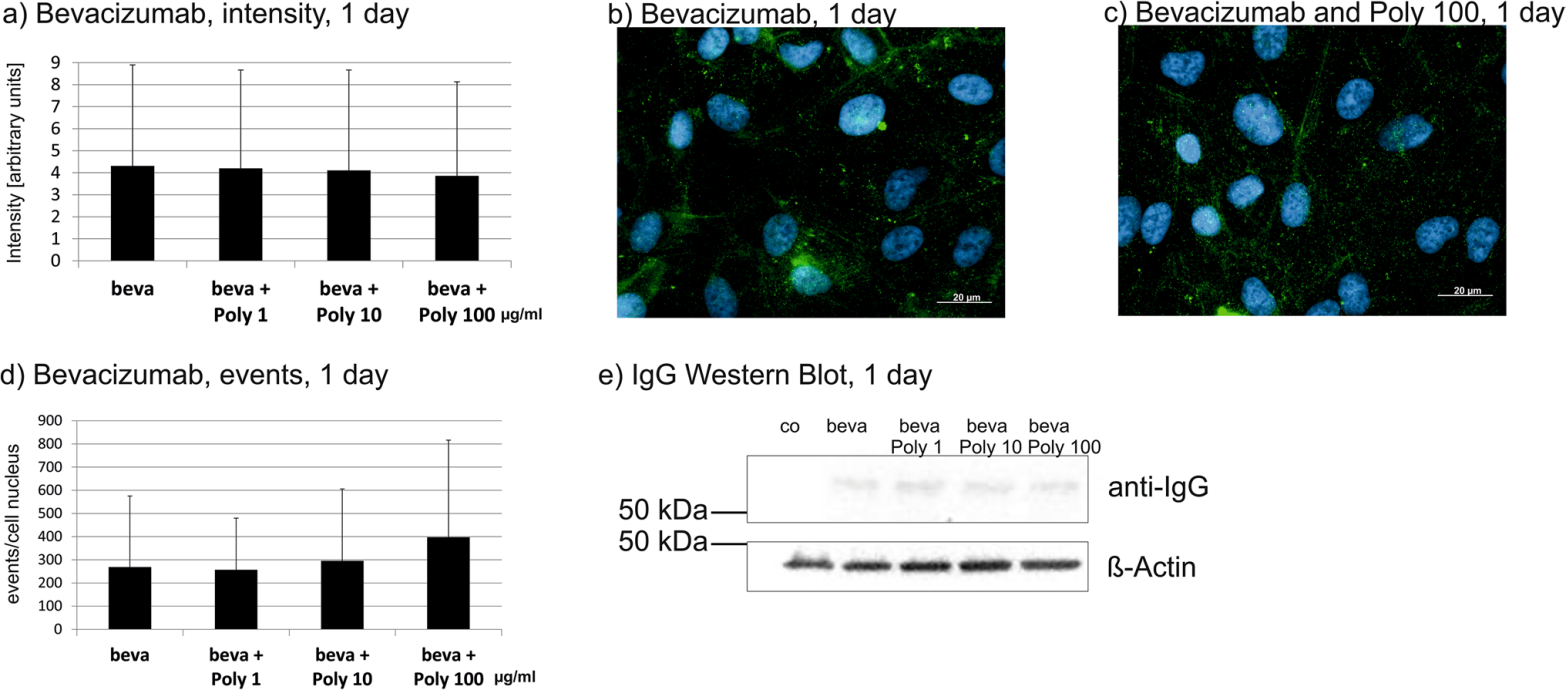
Intracellular bevacizumab signal in cells treated with 250 µg/ml bevacizumab and 1, 10, or 100 µg/ml Poly I:C for 1 day, displaying quantified intensity (**a**) and events per nucleus (**d**). Intensity or events of bevacizumab per cell nucleus were not changed after 1 day of incubation with Poly I:C. Example immunofluorescence pictures are given in (**b**) and (**c**); blue = nucleus; green = bevacizumab; example Western blot is shown in (**e**). Statistical significance for intensity and events evaluated with Student’s *t* test (*n* = 36). Abbreviations: beva = bevacizumab, Poly = polyinosinic:polycytidylic acid

**Fig. 3:**
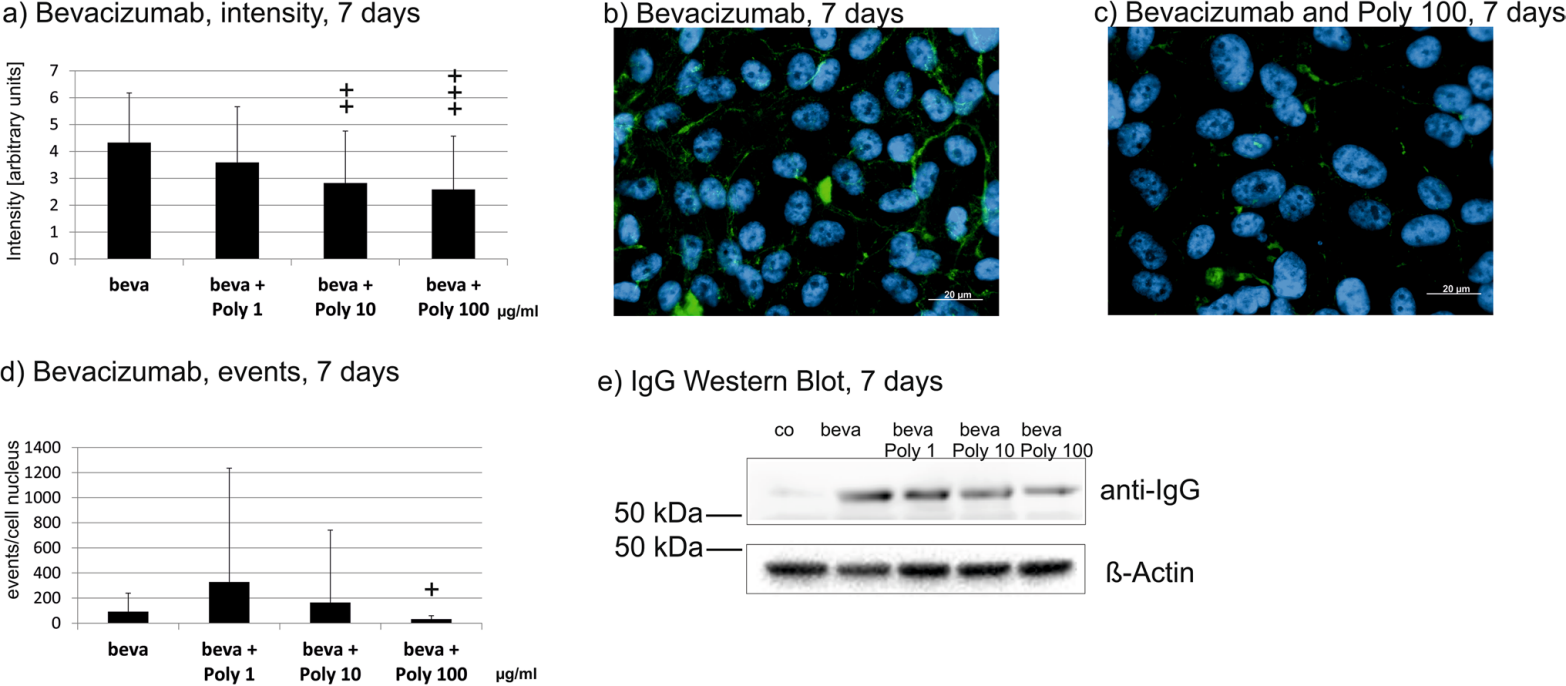
Intracellular bevacizumab signal in cells treated with 250 µg/ml bevacizumab and 1, 10, or 100 µg/ml Poly I:C for 7 days, displaying quantified intensity (**a**) and events per nucleus (**d**). Intensity was significantly reduced after 10 and 100 µg/ml Poly I:C, events of bevacizumab per cell nucleus were significantly reduced after 100 µg/ml Poly I:C. Example immunofluorescence pictures are given in (**b**) and (**c**); blue = nucleus, green = bevacizumab; example Western blot is shown in (**e**). Statistical significance for intensity and events evaluated with Student’s *t* test. + *p* < 0.05, +  + *p* < 0.01, +  +  + *p* < 0.001 compared to control (*n* = 36). Abbreviations: beva = bevacizumab, Poly = polyinosinic:polycytidylic acid

**Fig. 4:**
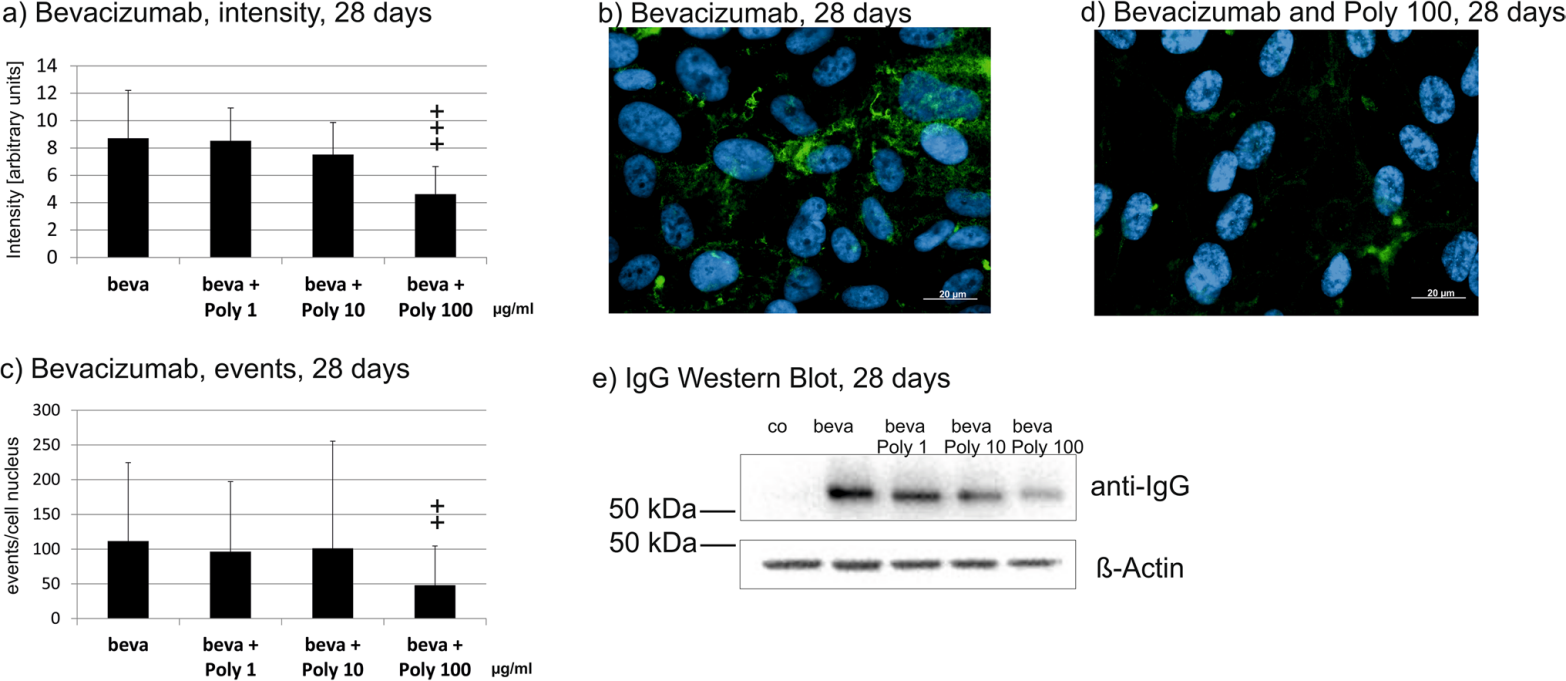
Intracellular bevacizumab signal in cells treated with 250 µg/ml bevacizumab and 1, 10, or 100 µg/ml Poly I:C for 28 days, displaying quantified intensity (**a**) and events per nucleus (**d**). Intensity and events per cell were significantly reduced after 100 µg/ml Poly I:C. Example immunofluorescence pictures are given in (**b**) and (**c**); blue = nucleus, green = bevacizumab; example Western blot is shown in (**e**). Statistical significance for intensity and events evaluated with Student’s *t* test. +  + *p* < 0.01, +  +  + *p* < 0.001 compared to control (*n* = 36). Abbreviations: beva = bevacizumab, Poly = polyinosinic:polycytidylic acid

Intracellular bevacizumab has been evaluated with immunofluorescence and quantitative analysis of the signal intensity. In addition, Western blots have been performed. Poly I:C did not influence intracellular bevacizumab intensity after 1 day of stimulation (Fig. 2a). Of note, intensity of intracellular bevacizumab after 1 day was partially not measurable, which is also reflected by a weak signal in Western blot (Fig. 2e). After 7 days, stimulation with 10 and 100 µg/ml Poly I:C reduced the signal significantly in immunofluorescence (Fig. 3a). An exemplary blot depicts similar findings in Western blot (Fig. 3e). Furthermore, 4 weeks of stimulation with 100 µg/ml Poly I:C reduced the signal significantly (Fig. 4a), again with an exemplary blot depicting a similar finding (Fig. 4e).

In addition, the events of intracellular bevacizumab were quantified. No influence on the number of bevacizumab events per cell nucleus was detected after 1 day of stimulation (Fig. 2d). After 7 days, the SD is very high after treatment with 1 or 10 µg/ml Poly I:C, but after treatment with 100 µg/ml, a clear and significant reduction in events per nucleus can be seen (Fig. 3d). After 4 weeks, again the treatment with 100 µg/ml Poly I:C significantly reduced the number of bevacizumab events in ARPE-19 cells (Fig. 4d).

Taken together, pro-inflammatory stimulation by Poly I:C reduces the uptake of bevacizumab in the RPE cells over time.

### Intensity actin filaments

**Fig. 5:**
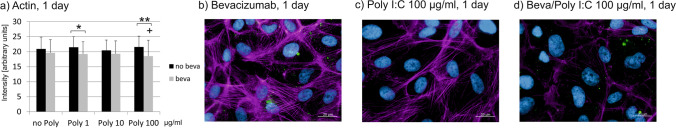
Intensity of actin signal treated with 250 µg/ml bevacizumab and 1, 10, or 100 µg/ml Poly I:C for 1 day (**a**). Co-treatment of bevacizumab with Poly I:C significantly reduced the actin signal compared to control at 100 µg/ml Poly I:C and compared to treatment with Poly I:C alone at 1 and 100 µg/ml Poly I:C. Example pictures are given in (**b**)–(**d**); purple = actin, blue = nucleus, green = bevacizumab. Statistical significance evaluated with Student’s *t* test. + *p* < 0.05 compared to control, *p < 0.05, **p < 0.01 comparing treatment with or without bevacizumab (*n* = 36). Abbreviations: beva = bevacizumab, Poly = polyinosinic:polycytidylic acid

After 1 day of treatment, the combined treatment with 100 µg/ml Poly I:C and bevacizumab significantly reduced the signal of actin compared to control. Also, the difference between cells treated with or without bevacizumab was significant for Poly I:C 100 µg/ml. In addition, the difference between treatment with or without bevacizumab for Poly I:C 1 µg/ml was significant (Fig. 5a).

**Fig. 6:**
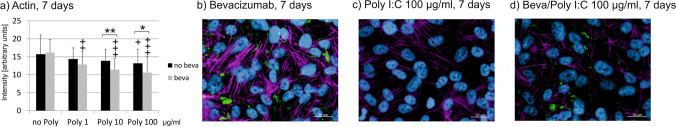
Intensity of actin signal treated with 250 µg/ml bevacizumab and 1, 10, or 100 µg/ml Poly I:C for 7 days (**a**). Co-treatment of bevacizumab with Poly I:C significantly reduced the actin signal compared to control at all concentrations of Poly I:C and compared to treatment with Poly I:C alone at 10 and 100 µg/ml Poly I:C. Example pictures are given in (**b**)–(**d**); purple = actin, blue = nucleus, green = bevacizumab. Statistical significance evaluated with Student’s *t* test. +  + *p* < 0.01, +  +  + *p* < 0.001 compared to control, *p < 0.05, **p < 0.01 comparing treatment with or without bevacizumab (*n* = 36). Abbreviations: beva = bevacizumab, Poly = polyinosinic:polycytidylic acid

**Fig. 7:**
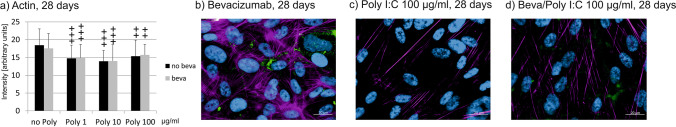
Intensity of actin signal treated with 250 µg/ml bevacizumab and 1, 10, or 100 µg/ml Poly I:C for 28 days (**a**). Treatment with Poly I:C with or without bevacizumab significantly reduced the actin signal compared to control at all concentrations. Example pictures are given in (**b**)–(**d**); purple = actin, blue = nucleus, green = bevacizumab. Statistical significance evaluated with Student’s *t* test. +  + *p* < 0.01, +  +  + *p* < 0.001 compared to control (*n* = 36). Abbreviations: beva = bevacizumab, Poly = polyinosinic:polycytidylic acid

After 7 days of treatment, treatment with Poly I:C alone significantly reduced the intensity of actin at a concentration of 100 µg/ml compared to untreated control. Combined treatment of bevacizumab with Poly I:C significantly reduced the signal of actin filaments compared to untreated control at all concentrations tested. The difference of treatment with or without bevacizumab is statistically significant for treatment with 10 and 100 µg/ml Poly I:C (Fig. 6a). After 4 weeks, treatment with Poly I:C with or without bevacizumab significantly reduces the intensity of actin. The differences between Poly I:C treatment with and without bevacizumab were not significant (Fig. 7a).

Taken together, the pro-inflammatory stimulation by Poly I:C influences the actin filaments of ARPE-19 cells and the effect is even stronger in combination with bevacizumab.

### Myosin 7a

**Fig. 8:**
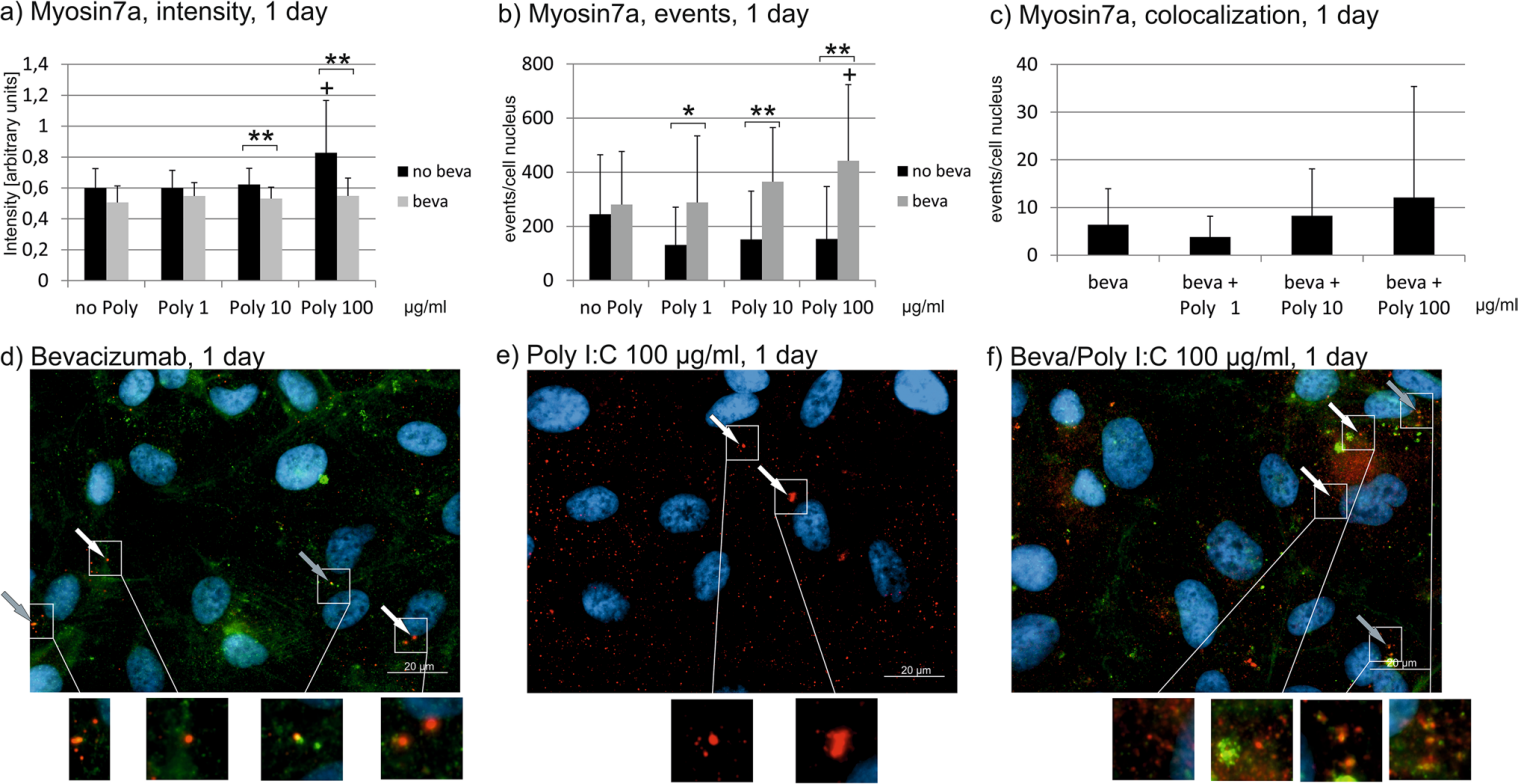
Myosin7a signal in immunofluorescence. Intensity (**a**), events (**b**), and colocalizations with bevacizumab (**c**) have been quantified after 1 day. Treatment with Poly I:C increases myosin intensity that is reduced by bevacizumab (**a**). Concerning the number of events of myosin7a, treatment with bevacizumab increases it compared to Poly I:C alone at all tested concentrations (**b**). Colocalization events between bevacizumab and myosin 7a are not changed by Poly I:C. Example pictures are given in (**d**)–(**f**); blue = nucleus, red = myosin7a, green = bevacizumab. White arrows: examples of myosin7a, gray arrows: examples of colocalizations; white squares indicate region with exemplary events shown in higher magnification. Statistical significance evaluated with Student’s *t* test. + *p* < 0.05 compared to control. *p < 0.05, **p < 0.01 comparing treatment with or without bevacizumab (*n* = 18). Abbreviations: beva = bevacizumab, Poly = polyinosinic:polycytidylic acid

**Fig. 9:**
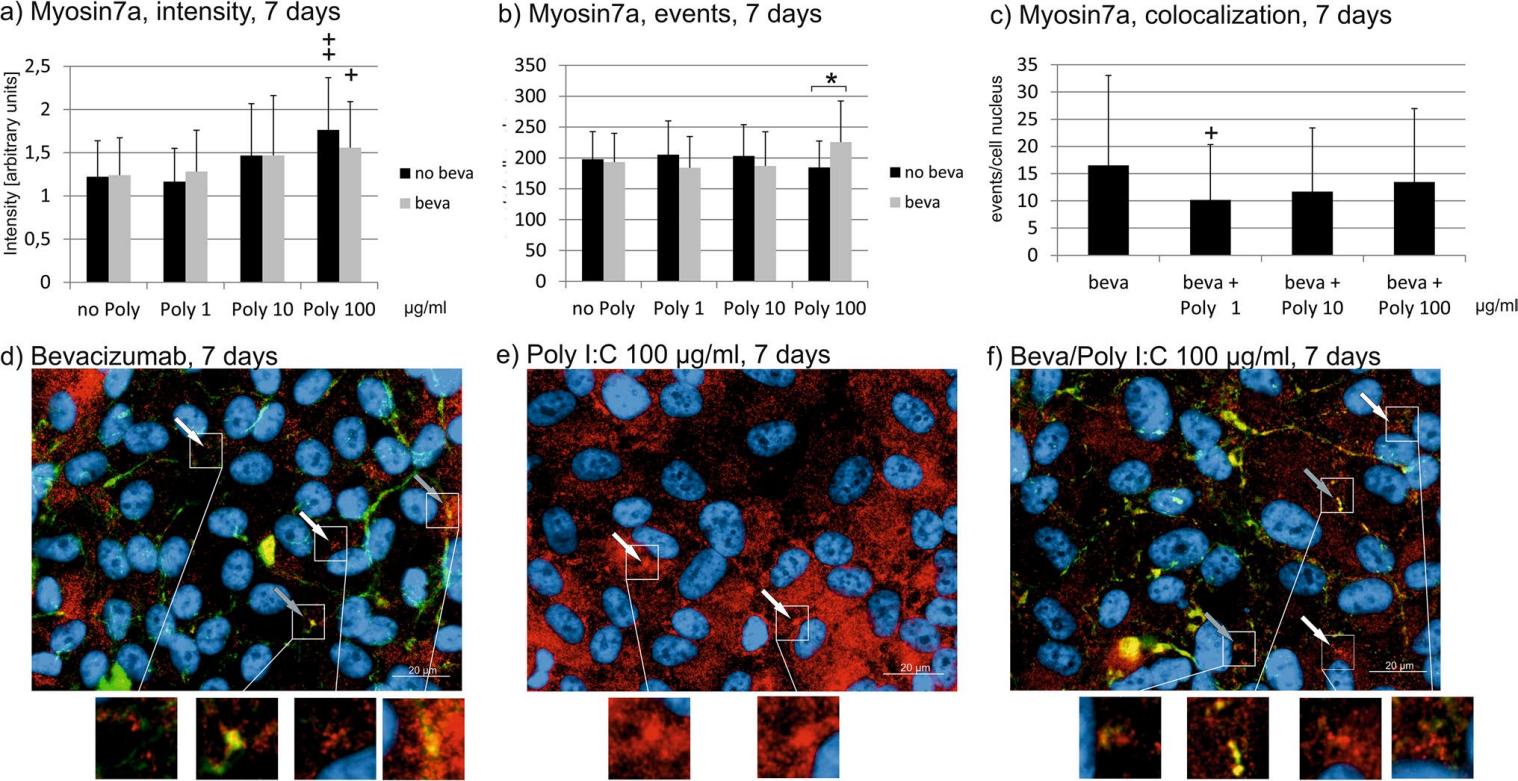
Myosin7a signal in immunofluorescence. Intensity (**a**), events (**b**), and colocalizations with bevacizumab (**c**) have been quantified after 7 days. Treatment with Poly I:C with or without bevacizumab increases myosin intensity at a concentration of 100 µg/ml (**a**). Concerning the number of events of myosin7a, treatment with bevacizumab increases it compared to Poly I:C alone at a concentration of 100 µg/ml Poly I:C (**b**). Colocalization events between bevacizumab and myosin 7a are decreased by 1 µg/ml Poly I:C. Example pictures are given in (**d**)–(**f**); blue = nucleus, red = myosin7a, green = bevacizumab. White arrows: examples of myosin7a, gray arrows: examples of colocalizations; white squares indicate region with exemplary events shown in higher magnification. Statistical significance evaluated with Student’s *t* test. + *p* < 0.05, +  + *p* < 0.01 compared to control. *p < 0.05 comparing treatment with or without bevacizumab (*n* = 18). Abbreviations: beva = bevacizumab, Poly = polyinosinic:polycytidylic acid

**Fig. 10:**
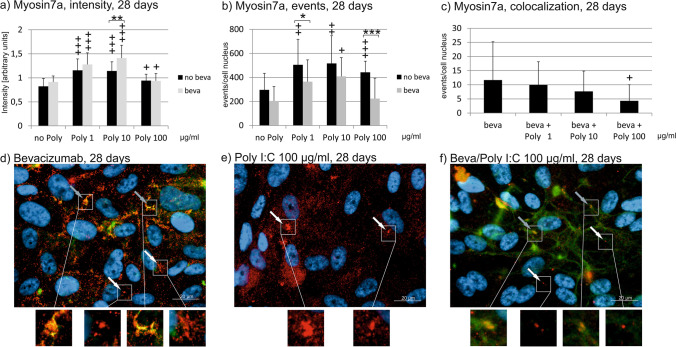
Myosin7a signal in immunofluorescence. Intensity (**a**), events (**b**), and colocalizations with bevacizumab (**c**) have been quantified after 28 days. Treatment with Poly I:C with or without bevacizumab increases myosin intensity all concentrations tested, with a combined treatment of bevacizumab and Poly I:C significantly elevating it compared to Poly I:C alone at a concentration of 10 µg/ml (**a**). Concerning the number of events of myosin7a, treatment with Poly I:C alone increases it at all concentrations tested, while a combined treatment with bevacizumab significantly reduces the event compared to Poly I:C alone at 1 and 100 µg/ml Poly I:C (**b**). Colocalization events between bevacizumab and myosin 7a are decreased by 100 µg/ml Poly I:C. Example pictures are given in (**d**)–(**f**); blue = nucleus, red = myosin7a, green = bevacizumab. White arrows: examples of myosin7a, gray arrows: examples of colocalizations; white squares indicate region with exemplary events shown in higher magnification. Statistical significance evaluated with Student’s *t* test. + *p* < 0.05, +  + *p* < 0.01, +  +  + *p* < 0.001 compared to control. *p < 0.05, **p < 0.01 comparing treatment with or without bevacizumab (*n* = 18). Abbreviations: beva = bevacizumab, Poly = polyinosinic:polycytidylic acid

After 1 day of treatment with 100 µg/ml Poly I:C, the intensity of myosin7a significantly increased, which was not seen when simultaneously treated with bevacizumab (Fig. 8a). Indeed, the differences between myosin7a intensity of cells treated with and without bevacizumab are significant for 10 and 100 µg/ml Poly I:C (Fig. 8a). After 7 days, stimulation with 100 µg/ml Poly I:C significantly increased myosin7a intensity with or without stimulation with bevacizumab (Fig. 9a). After 4 weeks of stimulation, treatment with Poly I:C of any concentration with or without bevacizumab increases the intensity of myosin7a. However, at a concentration of 10 µg/ml, this increase is significantly further elevated by treatment with bevacizumab (Fig. 10a).

Concerning myosin7a events, a significant difference for cells treated with Poly I:C with or without bevacizumab can be found after 1 day for all Poly I:C concentration, with bevacizumab increasing the number of events (Fig. 8b). The increase reaches significance compared to control at a concentration of 100 µg/ml Poly I:C with bevacizumab. After 7 days of incubation, the differences between treatment with or without bevacizumab is still significant at a concentration of 100 µg/ml Poly I:C (Fig. 9b). After 4 weeks, treatment with Poly I:C again increases the events of myosin7a; however, now the treatment with bevacizumab significantly reduces the events compared to treatment with Poly I:C seen at 1 and 100 µg/ml (Fig. 10b).

Concerning colocalization, treatment with Poly I:C for 1 day did not change the events of colocalization between bevacizumab and myosin7a at any concentration tested (Fig. 8c). After 7 days, however, Poly I:C at a concentration of 1 µg/ml significantly reduced the number of colocalizations between bevacizumab and myosin7a (Fig. 9c). After 4 weeks, the number of colocalizations between bevacizumab and myosin7a was significantly reduced when treated with 100 µg/ml Poly I:C (Fig. 10c).

Taken together, the treatment with Poly I:C seems to increase myosin7a, while long-term treatment with bevacizumab tends to reduce it. The colocalization between bevacizumab and myosin may be reduced by Poly I:C stimulation.

### Lamp2

**Fig. 11:**
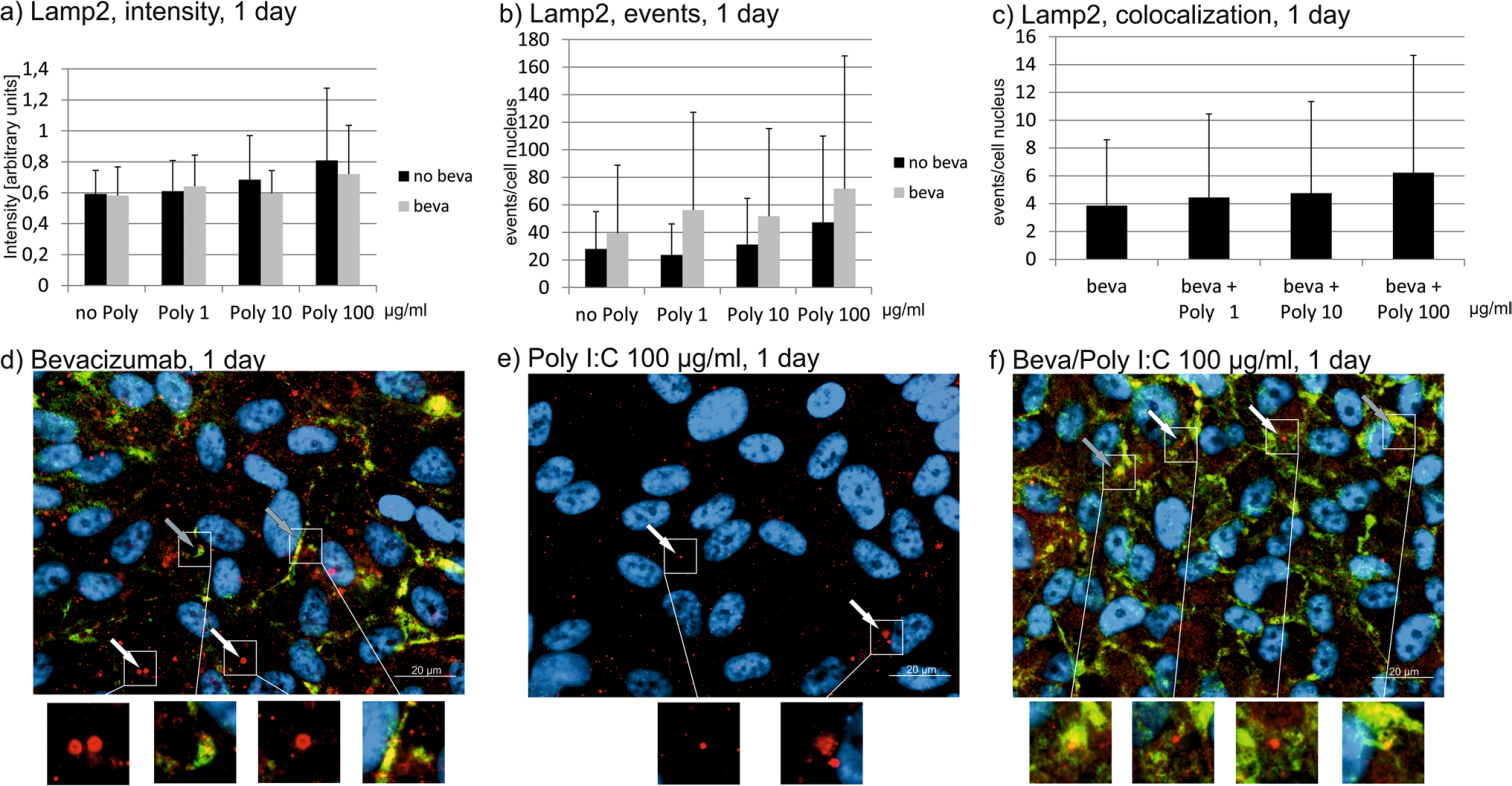
Lamp2 signal in immunofluorescence. Intensity (**a**), events (**b**), and colocalizations with bevacizumab (**c**) have been quantified after 1 day. No influence can be found on Lamp2 intensity, events, or colocalization. Example pictures are given in (**d**)–(**f**); blue = nucleus, red = Lamp2, green = bevacizumab. White arrows: examples of myosin7a, gray arrows: examples of colocalizations; white squares indicate region with exemplary events shown in higher magnification. Statistical significance evaluated with Student’s *t* test (*n* = 18). Abbreviations: beva = bevacizumab, Poly = polyinosinic:polycytidylic acid

**Fig. 12:**
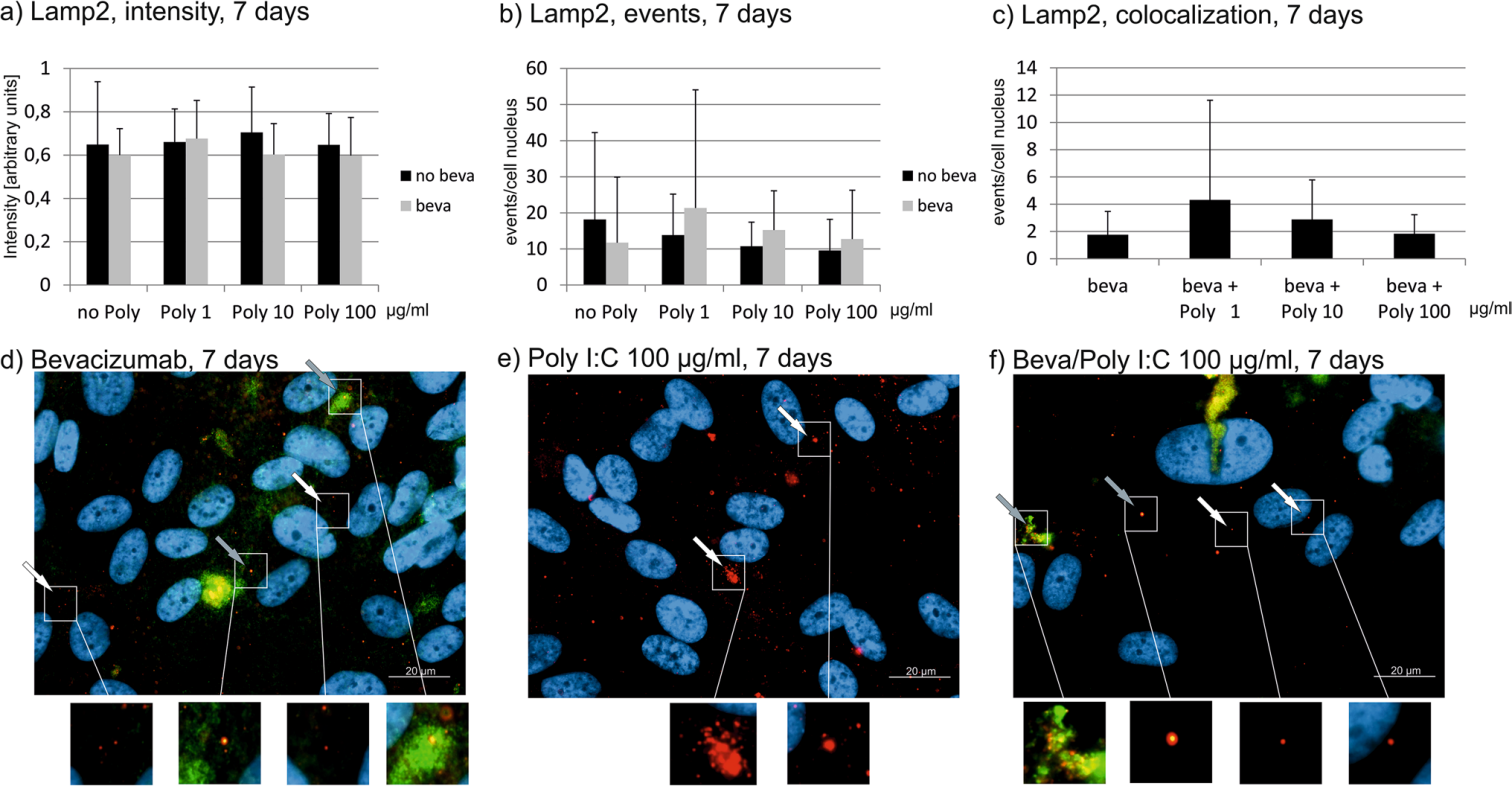
Lamp2 signal in immunofluorescence. Intensity (**a**), events (**b**), and colocalizations with bevacizumab (**c**) have been quantified after 7 days. No influence can be found on Lamp2 intensity, events, or colocalization. Example pictures are given in (**d**)–(**f**); blue = nucleus, red = Lamp2, green = bevacizumab. White arrows: examples of myosin7a, gray arrows: examples of colocalizations; white squares indicate region with exemplary events shown in higher magnification. Statistical significance evaluated with Student’s *t* test (*n* = 18). Abbreviations: beva = bevacizumab, Poly = polyinosinic:polycytidylic acid

**Fig. 13:**
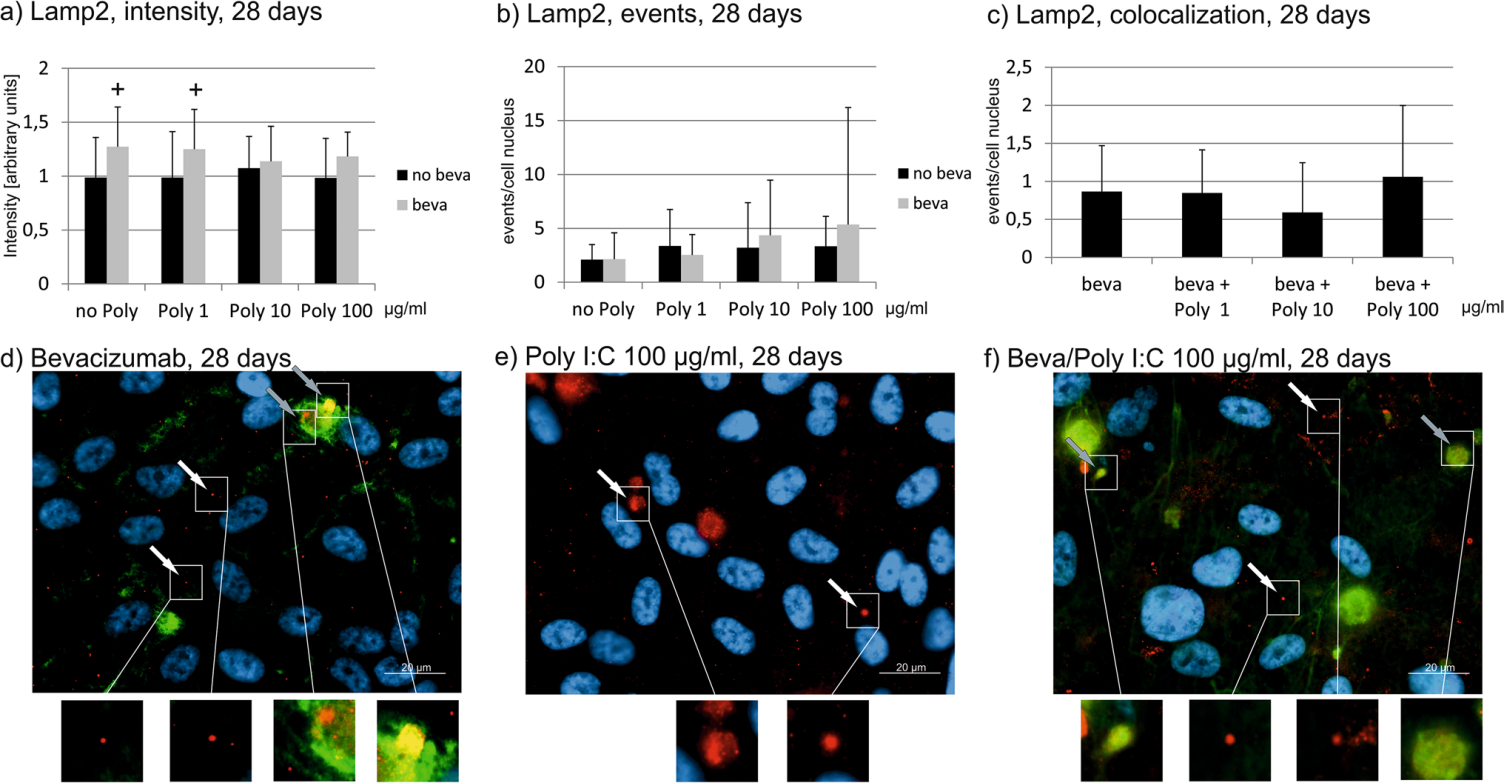
Lamp2 signal in immunofluorescence. Intensity (**a**), events (**b**), and colocalizations with bevacizumab (**c**) have been quantified after 7 days. Intensity significantly increases after treatment with bevacizumab alone and with bevacizumab and 1 µg/ml Poly I:C. No influence can be found on Lamp2 events or colocalization. Example pictures are given in (**d**)–(**f**); blue = nucleus, red = Lamp2, green = bevacizumab. White arrows: examples of myosin7a, gray arrows: examples of co-localizations; white squares indicate region with exemplary events shown in higher magnification. Statistical significance evaluated with Student’s *t* test. + *p* < 0.05 compared to control (*n* = 18). Abbreviations: beva = bevacizumab, Poly = polyinosinic:polycytidylic acid

The treatment with Poly I:C with or without bevacizumab does not affect the intensity Lamp2 staining in immunofluorescence after 1 day or after 7 days (Figs. 11a and 12a). After 4 weeks, treatment with bevacizumab alone and with bevacizumab combined with 1 µg/ml Poly I:C significantly increased the intensity of Lamp2 (Fig. 13a). The number of events are not significantly changed after 1 day of treatment with Poly I:C with or without bevacizumab (Fig. 11b). Similarly after 7 and 28 days of stimulation, the differences in events did not differ after treatment with Poly I:C with or without bevacizumab (Figs. 12b and 13b). In addition, the number of colocalizations between Lamp2 and bevacizumab per nucleus was evaluated. After 1 day, 7 days, or 4 weeks of stimulation, no differences in colocalizations could be found (Figs. 11c, 12c, and 13c).

Taken together, pro-inflammatory treatment with Poly I:C with or without bevacizumab shows little influence on Lamp2 expression or colocalization.

### Transepithelial transport of bevacizumab

**Fig. 14:**
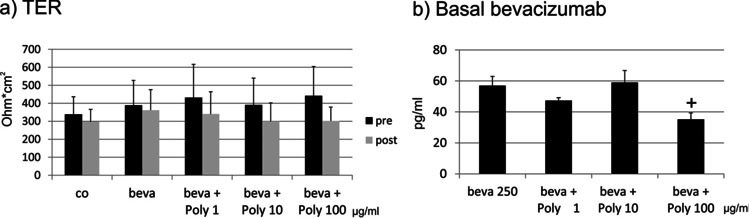
Transepithelial transport of bevacizumab in primary RPE cells. Transepithelial resistance of RPE cells before (pre) and 1 h after (post) apical application of 250 µg/ml bevacizumab (**a**), basal concentration of bevacizumab after 1 h of incubation, measured in ELISA (**b**). Treatment with bevacizumab with or without Poly I:C did not change the transepithelial resistance in RPE cells (**a**). The basal amount of bevacizumab was significantly reduced at a concentration of 100 µg/ml Poly I:C (**b**). Statistical significance evaluated with Student’s *t* test. + *p* < 0.05 compared to beva (*n* = 3). Abbreviations: beva = bevacizumab, Poly = polyinosinic:polycytidylic acid

We have investigated the transepithelial transport of bevacizumab in primary RPE cells, cultivated on transwell plates which showed a stable barrier, assessed by TER measurement. As ARPE-19 cells do not form a stable barrier, they are not a suitable model for this objective [34]. We have previously shown that the treatment with Poly I:C with the indicated concentrations (1, 10, and 100 µg/ml) alone has no influence on the TER of primary RPE cells [20]. TER was measured before (pre) and 1 h after (post) application of Poly I:C and/or bevacizumab. No significant differences were found between the TER pre- and post-treatment (Fig. 14a). Bevacizumab was added to the apical well of the transwell and the bevacizumab concentration was measured in the basal well after 1 h in an IgG ELISA. When treated with 100 µg/ml Poly I:C, the amount of bevacizumab found in the basal compartment was significantly reduced (Fig. 14b).

### Effect of bevacizumab on inflammatory cytokine secretion

**Fig. 15:**
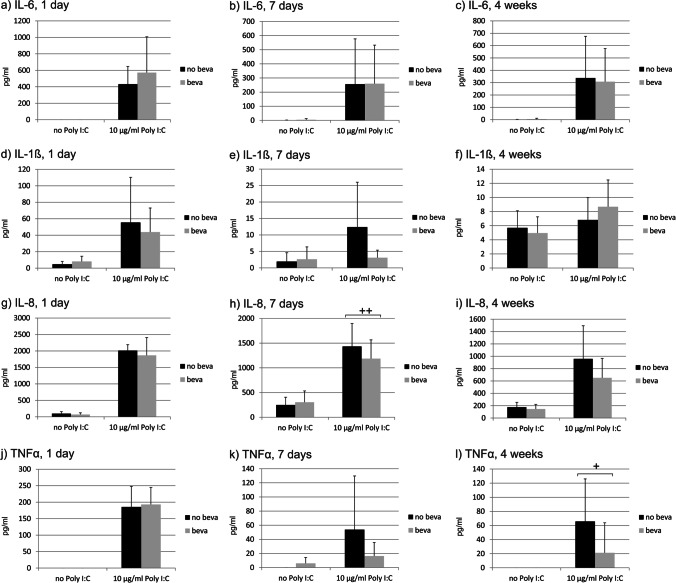
Influence of bevacizumab on the secretion of pro-inflammatory cytokines. Primary RPE cells were treated with 10 µg/ml Poly I:C for 1 day (**a**, **d**, **g**, **j**), 7 days (**b**, **e**, **h**, **k**), or 4 weeks (**c**, **f**, **i**, **l**), and the secretion of IL-6 (**a**–**c**), IL-1β (**d**–**f**), IL-8 (**g**–**i**), and TNFα (**j**–**l**) was evaluated in ELISA. No significant changes were found for IL-6 or IL-1β. Conversely, the secretion of IL-8 was significantly reduced by bevacizumab after 7 days (**h**) and the secretion of TNFα was reduced after 4 weeks (**l**). Statistical significance evaluated with Student’s *t* test between treated and non-treated with bevacizumab. + *p* < 0.05, +  + *p* < 0.01 (*n* = 3–5). Abbreviations: beva = bevacizumab, Poly = polyinosinic:polycytidylic acid

We have previously shown that RPE cells secrete cytokines in a time-dependent manner after Poly I:C stimulation [20]. Here, we tested the effect of bevacizumab on the release of IL-6, IL-1β, IL-8, and TNFα after stimulation with 10 µg/ml Poly I:C in primary porcine RPE. Bevacizumab did not increase the secretion of any of the tested cytokines in untreated cells at any time point tested (Fig. 15). In addition, bevacizumab did not alter the secretion of IL-6 (Fig. 15a–c) or IL-1β (Fig. 15d–f). For IL-8, bevacizumab significantly reduced its secretion after 7 days (Fig. 15h), and for TNFα, bevacizumab reduced the secretion after 4 weeks (Fig. 15l).

## Discussion

VEGF therapy has changed the treatment option of exudative AMD and has been a considerable improvement for the patients [9]. Nevertheless, VEGF antagonists have to be applied repeatedly, often over many years, and the initial gain of vision may be lost over time [35]. The effects of VEGF antagonists on the retina beyond inhibition of VEGF are not understood so far and in vitro studies that target repeated or long-term effects are scarce [36]. The application of bevacizumab in ophthalmology is common, irrespective of its off-label use [12]. We have shown before that bevacizumab is taken up by the RPE and interacts intracellularly, reducing phagocytosis and being transported along actin filaments via myosin [21, 24, 37]. Bevacizumab is transported through the epithelial barrier through this mechanism, reaching the subretinal space this way even where the RPE barrier function is still intact [21]. All experiments cited were done in healthy RPE cells. However, the AMD retina is not healthy and constantly activated by inflammation, as oxidative stress injuries, cell debris from degenerating cells, and cytokines from activated macrophages or microglia perpetuate a chronic pro-inflammatory state [18, 38, 39]. As pro-inflammatory stimuli have an impact on RPE function [20], this may also have an effect on its interaction with bevacizumab (or other VEGF antagonists). Therefore, in the present study, we have tested the effect of the pro-inflammatory TLR-3 agonist Poly I:C on the uptake, intracellular interaction, and transepithelial transport in RPE cells.

Our data show that long-term treatment with Poly I:C and bevacizumab may influence cell viability in the long term, as seen in trypan-blue exclusion assay and number of cell nuclei in ARPE-19 cells as well as in MTT in both ARPE-19 and primary RPE cells. The data suggest that the effect is mainly based on Poly I:C stimulation, which has been shown previously to reduce RPE viability [19, 20] and which was confirmed in the present study. Bevacizumab may accelerate this effect, as a significant reduction of viability could be found at selected time points and concentration in ARPE-19 cells in MTT, cell nuclei number, and trypan-blue exclusion assay. It should be noted, however, that the contrary effect was also seen, with an increase in cell nuclei in ARPE-19 or an increase in MTT signal in primary RPE seen at single time points and concentrations. Therefore, the combined effects of pro-inflammatory signaling and bevacizumab treatment could have an effect on RPE viability which should be investigated further. The uptake of bevacizumab may be an important pathway to transport the VEGF antagonist over the intact barrier to the subretinal space. In the present study, we quantified intracellular bevacizumab and could show that long-term treatment with Poly I:C reduced intracellular bevacizumab in medium-term (7 days) and long-term (4 weeks) treatment. Of note, the effect is not merely due to a reduced number of RPE cells in treatment, as not only the intensity but also the events of bevacizumab per cell nucleus are reduced. The reduced presence of bevacizumab is likely not to be due to an increased lysosomal degradation, as we see no increase in Lamp2 colocalization or expression. However, also an increased recycling of bevacizumab out of the cell could be possible. However, our data show a retarded, not accelerated, transport through the cells. Also, a reduced phagocytosis is not likely, as Poly I:C shows little influence on phagocytic activity in RPE cells [20]. As the uptake of bevacizumab is likely to be mediated via pinocytotic uptake in the endosomal pathway [24], pro-inflammatory signaling may alter this pathway. Further research is to be conducted to elucidate the influence of pro-inflammatory signaling on the function of RPE cells. As mentioned, the transepithelial transport of bevacizumab is also reduced under Poly I:C stimulation, which could be either a consequence of a reduced uptake or connected to the alterations seen after Poly I:C on the actin filaments and myosin7a colocalization. Indeed, the changes of the actin filaments under Poly I:C, which can be observed already after the first day of exposure and are significantly stronger when the pro-inflammatory stimulus are combined with bevacizumab, are intriguing as they suggest a change of cell morphology. In addition, the changes seem to display a transition from a more epithelial distribution (with submembrane actin filaments holding up the cell morphology) to a more fibroblastic appearance, with stress fibers becoming predominant. This may indicate a de-differentiation of the RPE cells, which could be of strong consequences in vivo. However, it has to be noted that these experiments have been conducted in ARPE-19 cells, which differ from primary RPE cells in many respects and which may dedifferentiate more easily than RPE cells in situ [34, 40].

A reduced uptake and a reduced transepithelial transport of bevacizumab could influence its efficacy in anti-VEGF treatment. Bevacizumab can interfere not only directly with VEGF, but is also able to reduce VEGF expression intracellularly [28]. It has to be noted, however, that the mechanism of this influence on expression is so far not understood and may well be mediated via an interference with an autocrine VEGF–VEGFR-2 amplification loop, independent of intracellular bevacizumab uptake [41]. In exudative AMD, choroidal neovascularizations (CNV) which need to be treated by VEGF antagonists can be found in the retina, in the subretinal space, or beneath the RPE [4]. In the case of the lesion being beneath the RPE (and presuming that the RPE barrier is intact), the VEGF antagonists would have to be transported through the barrier layer [25]. Of note, the present study and previously published data have shown that the barrier of the RPE cells in our experimental system is still functioning after 1 h of stimulation with Poly I:C [20]; therefore, the transepithelial transport of the intact barrier can be mimicked here. A longer incubation with Poly I:C, however, may interfere with the barrier function [27]. The reduced transepithelial transport of bevacizumab under inflammatory stimuli may therefore indeed reduce the amount of bevacizumab available beneath the RPE. The combined effect was only found when the highest concentration of Poly I:C was used, suggesting a concentration-dependent effect, which may exceed the physiological response. Furthermore, if the barrier was already breached, this effect in the clinic on bevacizumab efficacy would be negligible.

In addition to potential effects of Poly I:C on bevacizumab, our data also show (modest) effects of bevacizumab on the pro-inflammatory cytokine release induced by Poly I:C. Interestingly, the secretion of IL-8 (after 7 days) and of TNFα (after 4 weeks) is reduced. IL-8 is elevated in the aqueous humor of wet AMD patients and considered to be pro-angiogenic [42, 43]; therefore, its reduction by bevacizumab may contribute to its anti-angiogenic properties under pro-inflammatory conditions. TNFα, on the other hand, is considered to be neurotoxic [44]. Moreover, long-term TNFα stimulation has been shown to abolish the expression of RPE65 in RPE cells, a protein important for the recycling of the visual pigment [20]. Therefore, a reduction of TNFα may be beneficial for photoreceptor function.

Taken together, the present study shows that pro-inflammatory stimuli affect the uptake, intracellular pathways, and transepithelial transport of bevacizumab which could be of consequence for bevacizumab availability and efficacy. On the other hand, bevacizumab may have a (regulatory) influence on pro-inflammatory cytokine secretion. Further research is warranted to elucidate the effects of inflammation in the interaction of RPE cells with anti-VEGF compounds.

## Data Availability

The data are available upon request.
